# Conidia Fusion: A Mechanism for Fungal Adaptation to Nutrient-Poor Habitats

**DOI:** 10.3390/jof9070755

**Published:** 2023-07-17

**Authors:** Xinju Yang, Fa Zhang, Yaoquan Yang, Faping Zhou, Saranyaphat Boonmee, Wen Xiao, Xiaoyan Yang

**Affiliations:** 1 Institute of Eastern-Himalaya Biodiversity Research, Dali University, Dali 671003, China; yxj2002157@dingtalk.com (X.Y.); zhangf@eastern-himalaya.cn (F.Z.); yangyq@eastern-himalaya.cn (Y.Y.); zhoufaping@eastern-himalaya.cn (F.Z.); xiaow@eastern-himalaya.cn (W.X.); 2 Center of Excellence in Fungal Research, Mae Fah Luang University, Chiang Rai 57100, Thailand; saranyaphat.boo@mfu.ac.th; 3 School of Science, Mae Fah Luang University, Chiang Rai 57100, Thailand; 4 Collaborative Innovation Center for Biodiversity and Conservation in the Three Parallel Rivers Region of China, Dali 671003, China; 5 The Provincial Innovation Team of Biodiversity Conservation and Utility of the Three Parallel Rivers Region, Dali University, Dali 671003, China; 6 Yunling Back-and-White Snub-Nosed Monkey Observation and Research Station of Yunnan Province, Dali 671003, China; 7 Key Laboratory of Yunnan State Education Department on Er’hai Lake Basin Protection and the Sustainable Development Research, Dali University, Dali 671003, China

**Keywords:** conidia fusion body, environmental adaptation, nematode-trapping fungi, nutrient concentration, Orbiliomycetes

## Abstract

Conidia fusion (CF) is a commonly observed structure in fungi. However, it has not been systematically studied. This study examined 2457 strains of nematode-trapping fungi (NTF) to explore the species specificity, physiological period, and physiological significance of CF. The results demonstrated that only six species of *Arthrobotrys* can form CF among the sixty-five tested NTF species. The studies on the model species *Arthrobotrys oligospora* (DL228) showed that CF occurred in both shed and unshed plus mature and immature conidia. Additionally, the conidia fusion rate (CFR) increased significantly with the decrease of nutrient concentration in habitats. The studies on the conidia fusion body (CFB) produced by *A. oligospora* (DL228) revealed that the more conidia contained in the CFB, the faster and denser the mycelia of the CFB germinated in weak nutrient medium and soil plates. On the one hand, rapid mycelial extension is beneficial for the CFB to quickly find new nutrient sources in habitats with uneven nutrient distribution. On the other hand, dense mycelium increases the contact area with the environment, improving the nutrient absorption efficiency, which is conducive to improving the survival rate of conidia in the weak nutrient environment. In addition, all species that form CF produce smaller conidia. Based on this observation, CF may be a strategy to balance the defects (nutrient deficiency) caused by conidia miniaturization.

## 1. Introduction

Soil is the largest repository of microorganisms in nature, hosting billions of microorganisms per gram [1,2]. However, the survival resources contained in the soil are limited, especially the essential nutrients for microbial survival, which are scarce and unevenly distributed [3]. In this situation, soil microorganisms face enormous nutritional competition pressure, especially for those single-cell microorganisms and the propagules of multicellular microorganisms that are relatively independent and carry fewer nutrients [4,5,6]. Under these circumstances, microorganisms have evolved various structures to adapt to such nutrient-poor soil environments [5,6,7,8,9,10].

Conidia fusion (CF) is a phenomenon where conidia form tube-like structures to connect multiple originally independent conidia into a single entity [11,12,13]. This structure has been described in over 70 fungal species, and its physiological significance has been explored to varying degrees in species such as *Colletotrichum lindemuthianum*, *Fusarium oxysporum*, *Neurospora crassa*, and *Epichloe festucae* [14,15] since its first report by Tulasne et al. in 1863 [16]. Roca et al. (2003) proposed that the physiological significance of CF may be to facilitate nuclear exchange between multiple originally independent conidia by connecting them [17], and the process of the nuclear exchange between fused conidia was confirmed by Ishikawa et al. (2010) using confocal microscopy live cell imaging and fluorescence chromo-genesis techniques [18]. In addition, many studies have shown that the formation of CF is obviously species specificity and closely related to the nutrient content and composition of the habitat [19,20,21,22]. For example, the conidia of *Colletotrichum lindemuthianum* does not fuse in nutrient-rich medium but only in nutrient-poor water agar medium [20]; *Leptosphaeria coniothyrium*, *Sclerotinia fructigena*, *Botrytis* sp., and *Fusarium* sp. produce more fused conidia in nutrient-poor medium but lower fusions in nutrient-rich medium [21]; *Fusarium oxysporum* does not fuse in water agar or PDB medium but has a higher conidia fusion rate (CFR) in 1% PDB + 25 mM NaNO_3_ medium [22]. Although the CFR of different species or strains had different responses to the nutrient content and composition of the habitat, the rule that the CFR was inversely proportional to the nutrient concentration of the habitat was confirmed for almost all species. Based on the above research, scholars speculated that another significance of CF might be an adaptation to nutrient deficiency in the habitat [23]. However, this hypothesis has not been supported by direct experimental data, and the way CF adapts to nutrient-poor environments is unclear. Moreover, previous studies have only focused on a few or even a single species in a specific group of fungi [12,14,17,19,20], resulting in an insufficient understanding of the significance of CF in the evolution of related groups.

Nematode-trapping fungi (NTF) in Orbiliomycetes are a highly specialized group of ancient monophyletic fungi that can form various trapping structures to capture nematodes [24,25,26]. Taxonomically, all Orbiliomycetes NTF have been divided into *Arthrobotrys* (catches nematodes with adhesive networks), *Dactylellina* (produces adhesive knobs, adhesive branches, and non-constricting rings), and *Drechslerella* (captures nematodes using constricting rings) genera based on their trapping structures according to modern molecular biology research [27,28,29]. This group of fungi has become an ideal and key group for the biological control of parasitic nematodes and for studying fungal adaptive evolution because of their unique nematode-trapping function and morphological structures [30,31]. Additionally, it also has gradually become a model group for studying fungal physiology due to its mature research methods, moderate group size (3 genera, 119 species), unique survival strategy, and diversified morphological structure (predation structure and conidia) [32,33]. CF is also a common structure in NTF. However, CF has not been systematically studied, except for only two brief studies which indicated that the CFR of *A. oligospora* can reach 1%–2% [34,35]. In addition, NTF is an ideal group for the systematic study of CF because the types of CF in NTF are diverse (two-conidia fusion, three-conidia fusion, four-conidia fusion, etc.).

Based on the above, this study focuses on NTF and conducts a systematic study on CF to clarify the species specificity and physiological period, as well as to explore the physiological and evolutionary significance of CF. The results will deepen our understanding of fungi adaptation strategy and contribute to the in-depth exploration of NTF evolution.

## 2. Materials and Methods

### 2.1. Experimental Materials

#### 2.1.1. Testing Nematode-Trapping Fungi (NTF)

All Orbiliomycetes NTF strains involved in this study were provided by the Germplasm Resources Center of the Institute of Eastern-Himalaya Biodiversity Research, including 2457 NTF strains (3 genera, 65 species) isolated from soil and freshwater sediment samples from Yunnan, China (Table S1, Figure S1).

#### 2.1.2. Culture Medium

Corn meal agar (CMA) [36] plates with different nutrient concentrations: Boiling 30 g, 20 g, 10 g, and 0 g corn meal in 800 mL of distilled water for 20 min and filtering the mixtures through four layers of gauze, supplementing the filtrates to 1000 mL with distilled water, and adjusting the pH to 7.2 using pH meter (Ohaus Instruments Company, Limited, Shanghai, China), then adding 18 g of agar, respectively. Autoclaving at 121 °C for 30 min (Dongya Pressing Vessel Manufacturing Company, Limited, Shanghai, China), pouring the mixtures into 60 mm plates to create CMA plates with nutrient concentrations of 3%, 2%, 1%, and 0%.

Corn meal liquid medium (CM) with different nutrient concentrations: The preparation method was the same as the CMA plates above but without agar.

Soil plates: The field soil (1000 g) was collected from a depth of 0–15 cm from the Agronomy Practice and Teaching Base of Dali University (100°10′30″ E, 25°41′04″ N). The large particles in the soil were sieved out using a 20-mesh sieve (Haoquan screen factory, Shaoxing, China). The remaining soil (500 g) was mixed and autoclaved at 121 °C for 30 min (Dongya Pressing Vessel Manufacturing Company, Limited, Shanghai, China). Then, 20 g of sterile soil was put in a sterile 60 mm plate and moistened with 3 mL of sterile water.

### 2.2. Experimental Methods

#### 2.2.1. Strain Rejuvenation

The testing strains were picked out from the frozen tubes using sterile toothpicks and incubated on 3% CMA plates at 28 °C in the dark.

#### 2.2.2. Species Specificity of Conidia Fusion (CF)

Screening of CF-formation species: The revived strains were transferred to three fresh 3% CMA plates with sterile toothpicks and incubated at 28 °C in the dark. Once they entered the full conidia productive period (from the 4th day after inoculation, a stereomicroscope was used to observe and rough estimate the conidia number of the strain every two days, when the conidia number did not increase significantly, it was the full conidia productive period of the strain. The full conidia productive period of *Arthrobotrys* species is usually between the 6th and 10th day, while for *Dactylellina* and *Drechslerella* species it is usually between the 8th and 15th day). The conidia were taped with 1 cm × 1 cm scotch tape to make the temporary slides. These slides were observed under an ordinary microscope (Optec Instrument Company, Limited, Chongqing, China) for the presence of CF (three slides per plate).

Conidia fusion rate (CFR) of CF-formation species: For each CF-formation species, three strains were inoculated in 3% CMA medium at 28 °C in the dark (three plates per strain). When they entered the full conidia productive period (6th–8th day), 1.5 mL sterile saline solution was added to the plate and the conidia were fully eluted. The conidia eluent was collected in a 1.5 mL sterile centrifuge tube and centrifuged at 2300 r/min for 5 min. The supernatant fluid was removed and 500 μL concentrated conidia suspension was retained and mixed thoroughly. Subsequently, 10 μL concentrated conidia suspension was taken to make temporary slides (three slides per plate) and the total number of conidia and fused conidia in each slide were counted under a microscope. The CFR of each plate = The total number of fused conidia in the three slides/Total number of conidia in the three slides × 100%. The average CFR of the three plates for each strain is the CFR of the strain.

#### 2.2.3. Physiological Period of CF Formation

*A. oligospora* (DL228), a model species of NTF [37], and a representative species of CF was used for subsequent studies.

To clarify whether CF occurred when the conidia are immature or mature, *A. oligospora* (DL228) was inoculated on 3% CMA plates at 28 °C in the dark. When the full conidia productive period (7th day) was reached, the conidia were taped with 1 cm × 1 cm scotch tape to make temporary slides and observed under a microscope. The presence of complete septa of the conidia was used as the criterion to determine whether the conidia were mature [34].

To determine whether the conidia would fuse after shedding from the conidiophores, *A. oligospora* (DL228) was inoculated on 3% CMA plates at 28 °C in the dark. When the full conidia productive period (7th day) was reached, 1.5 mL sterile saline solution was added to each plate, and the conidia were fully eluted. The conidia eluent was collected into a sterile centrifuge tube (40 mL). Most conidia fusion bodies (CFBs) were removed by filtration through eight layers of lens wiping paper and eight layers of 500 mesh gauze. The filtrate was collected and centrifuged at 2300 r/min for 3 min to retain 200 μL concentrated conidia suspension. The original CFR of conidia was counted using the method in Section 2.2.2. Afterwards, 10 μL concentrated conidia suspensions were loaded into PCR tubules preloaded with 10 μL of 0%, 1%, 2%, and 3% CM (three tubes per nutrient concentration), respectively. After incubation for 12 h at 28 °C in the dark, all the liquid in the tubes was used to make temporary slides, and the CFR was calculated for each nutrient concentration using the method in Section 2.2.2.

#### 2.2.4. Effect of Nutrient Concentration on CFR

*A. oligospora* (DL228) was incubated in 0%, 1%, 2%, and 3% CMA plates at 28 °C in the dark (three plates per nutrient concentration). When they entered the full conidia productive period (7th day), 1.5 mL sterile saline solution was added to each plate, and the conidia were fully eluted. The conidia eluent was collected in a 1.5 mL sterile centrifuge tube and centrifuged at 2300 r/min for 5 min. 500 μL concentrated conidia suspension was retained and mixed thoroughly. The CFR of *A. oligospora* (DL228) at each nutrient concentration was counted using the method described in Section 2.2.2.

#### 2.2.5. Effect of Conidia Number in Conidia Fusion Body (CFB) on the Germination of the CFB

*A. oligospora* (DL228) was inoculated on 3% CMA plates. Conidia were eluted with 1.5 mL sterile saline solution. 200 μL conidia suspension was dispersed uniformly on the surface of the 0% CMA plates. The plates were observed under a stereomicroscope. The single conidium, two-conidia, and three-conidia CFBs were picked out and transferred to the surface of 0% CMA plates with a sterile inoculating needle and incubated at 28 °C in the dark, respectively (three plates for each type of CFB). The plates inoculated with CFBs were upside down on the stage of a stereomicroscope to observe the germination and growth of the CFBs, and the hypha vein of CFB germination were depicted using a marker on the bottom of the plate on the third, sixth, and ninth day, respectively.

At the same time, single conidium, two-conidia, and three-conidia CFB were picked out and transferred to the center of the soil plates using the same methods and incubated at 28 °C in the dark (three plates for each type of CFB). The extension length of the mycelium of the different CFBs were measured under a stereomicroscope on the sixth and ninth day, respectively.

#### 2.2.6. Data Management and Analysis

In order to determine the effect of nutrient concentration on the CFR of *A. oligospora* (DL228) and the effect of the conidia number contained in the CFB on their germination in the soil, the differences between the treatment groups in each dataset were compared in pairs, respectively, using the following methods (taking the effect of nutrient concentration on the CFR of *A. oligospora* (DL228) as an example): (1) SPSS (version 17; SPSS Inc., Armonk, NY, USA) was used to perform the Shapiro-Wilk test to check the normality of data distribution. When the *p* value exceeds 0.05, the dataset conforms to the normal distribution and vice versa. (2) One-way ANOVA test in SPSS (version 17; SPSS Inc., Armonk, NY, USA) was used to test the variance homogeneity of the dataset. When the *p* value exceeds 0.05, the dataset conforms to variance homogeneity and vice versa. (3) For a dataset that satisfies both normal distribution and homogeneity of variance, the one-way ANOVA was performed using SPSS (version 17; SPSS Inc., Armonk, NY, USA) to clarify the differences in the dataset. The Tukey Honest Significance Difference Test (HSD, 5% precision level) in SPSS (version 17; SPSS Inc., Armonk, NY, USA) was used further to determine the pairwise differences between treatment groups. GraphPad Prism 7.00 was used to manage the experimental data and draw the bar charts with mean values and standard deviation (SD).

## 3. Results

### 3.1. Species Specificity of Conidia Fusion (CF)

**Figure 1:**
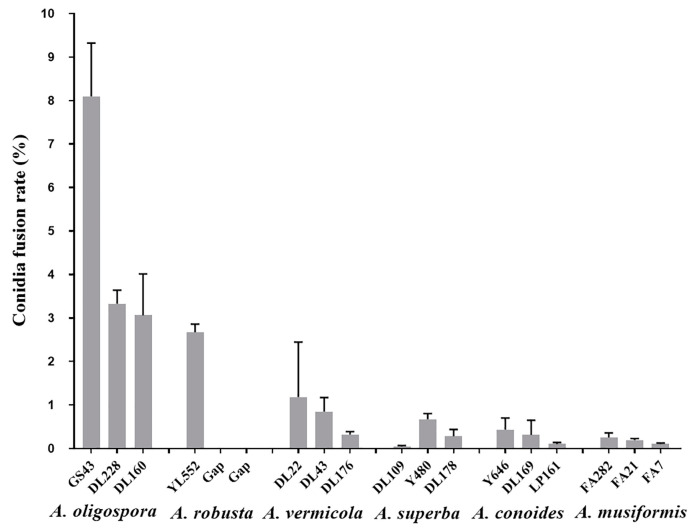
CFR of six CF-formation species. Values are the mean ± SD (*n* = 3). The number above the species name in the horizontal coordinate indicates the strain number. There is only one strain for *A. robusta* in our laboratory. The missing strains are labeled with gap.

After the screening, six *Arthrobotrys* species (*A. oligospora*, *A. superba*, *A. vermicola*, *A. musiformis*, *A. conoides,* and *A. robusta*) among sixty-five tested NTF species produced CF. The conidia fusion rate (CFR) of different strains of the same species are different, but the CFR of *A. oligospora* is generally higher (3.06% ± 0.95–8.10% ± 1.22) (Figure 1).

### 3.2. Physiological Period of CF

**Figure 2:**
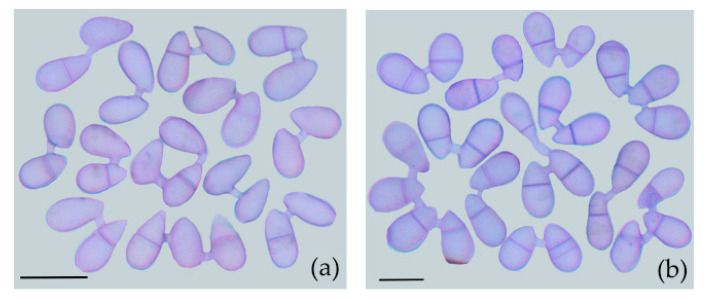
CF of *A. oligospora* (DL228). (**a**) CF occurred in non-septate conidia; (**b**) CF occurred in one-septate conidia. Scale = 20 µm.

Observations of CF produced by *A. oligospora* (DL228) showed that CF occurred in both one-septate and non-septate conidia, indicating that both mature and immature conidia fused (Figure 2).

**Figure 3:**
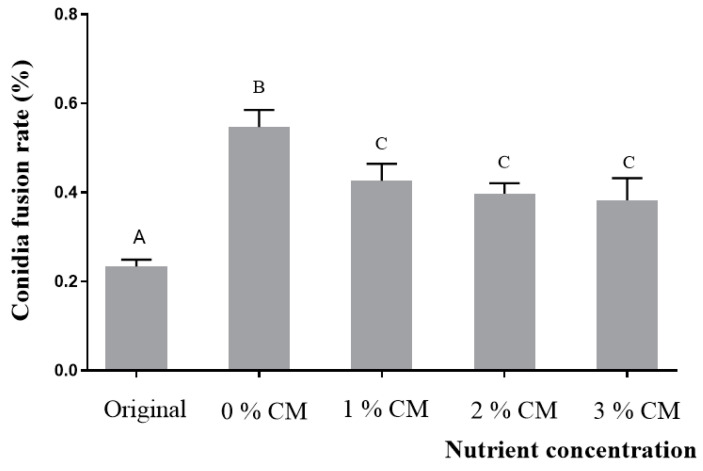
CFR of exfoliated conidia (*A. oligospora*, DL228) in the liquid media with different nutrient concentrations. Values are the mean ± SD (*n* = 3). The capital letters indicate significant differences among different treatment groups.

Statistical analysis showed that the CFR dataset of conidia after elution, filtration, and culture for 12 h satisfied both normal distributed (*p* = 0.587) and variance homogeneity (*p* = 0.429). One-way ANOVA results showed that there are significant differences in the dataset (F (4, 14) = 29, *p* < 0.0001). The CFRs of all the conidia after elution, filtration, and culture for 12 h were significantly higher than that without culture (*p* < 0.0001), indicating that the conidia also can fuse after shedding. Additionally, the CFR cultured in 0% CM medium was significantly higher than in other nutrient concentration media (*p* < 0.014) (Figure 3).

### 3.3. CFR of A. oligospora (DL228) Cultured at Different Nutrient Concentrations

**Figure 4:**
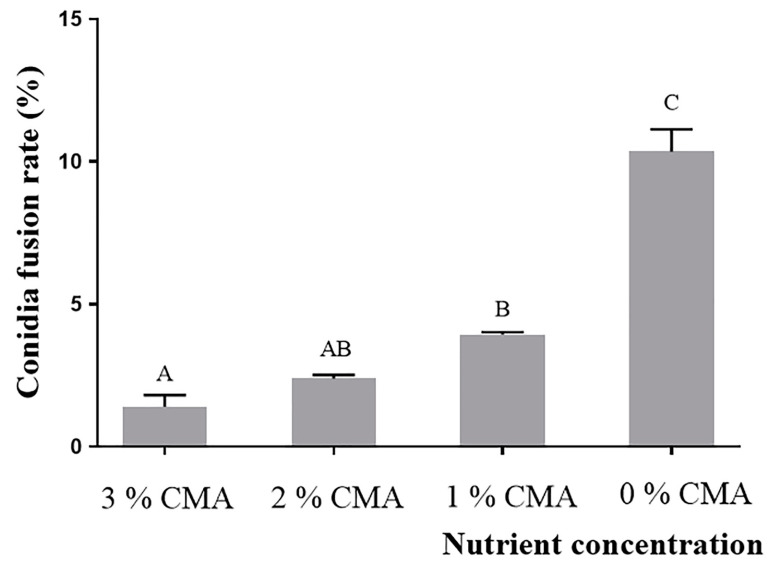
The CFR of *A. oligospora* (DL228) at different nutrient concentrations. Values are the mean ± SD (*n* = 3). The capital letters indicate significant differences among different treatment groups.

The normal distribution and variance homogeneity test showed that the datasets of CFR of *A. oligospora* (DL228) cultured in the CMA medium with different nutrient concentrations met the normal distribution (*p* = 0.055) and chi-squared (*p* = 0.081). One-way ANOVA showed that the CFR of *A. oligospora* (DL228) differed significantly (F (3, 11) = 71, *p* < 0.0001) at different nutrient concentrations of CMA medium and gradually increased with the decrease of nutrient concentration. The CFR of *A. oligospora* (DL228) in 0% CMA medium had the highest CFR of 10.3 ± 0.14% (Figure 4).

### 3.4. Influence of Conidia Number in Conidia Fusion Body (CFB) on the Germination of CFB

**Figure 5:**
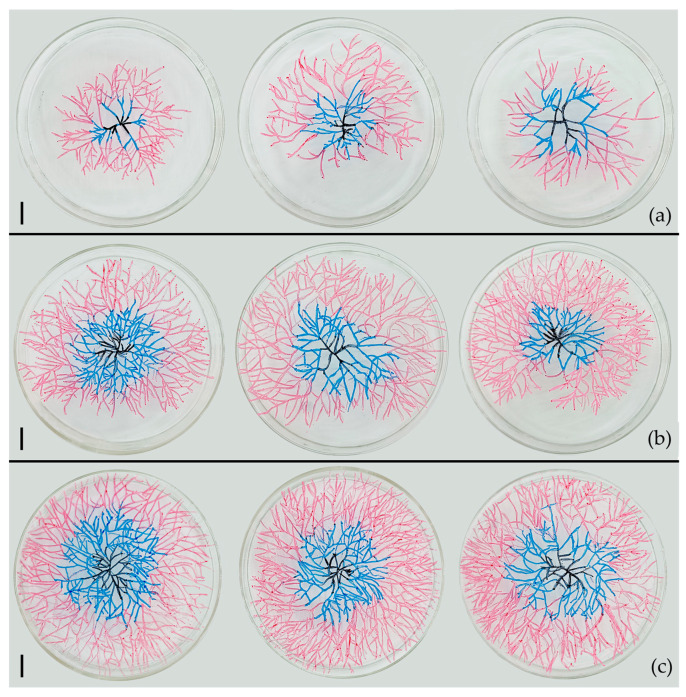
Germination and growth of CFBs (*A. oligospora,* DL228) with different conidia numbers in 0% CMA medium. (**a**) Germination of single conidium; (**b**) Germination of two-conidia CFBs; (**c**) Germination of three-conidia CFBs. Three plates in the same row represent three replicates of each type of CFB. The black, blue, and red lines mark the mycelial growth venation on the third, sixth, and ninth days, respectively. Scale bar = 1 cm.

In 0% CMA medium, as the number of conidia contained in the CFB increased, the mycelium sprouting from the CFB extended more rapidly and densely (Figure 5).

**Figure 6:**
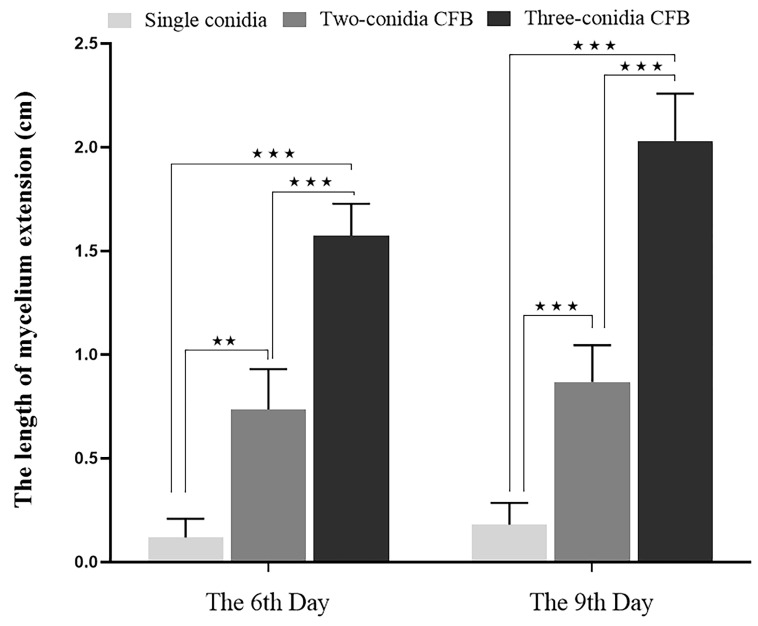
The mycelial extension length of CFBs (*A. oligospora,* DL228) with different conidia numbers in soil plates. Values are the mean ± SD (*n* = 3). ★★ indicates a significant difference between the treatment groups (0.01 < *p* < 0.05), ★★★ indicates an extremely significant difference between the treatment groups (*p* < 0.01).

The datasets on the effect of the conidia number contained in the CFB on the extension of the mycelium in soil (6th and 9th day) were tested to satisfy a normal distribution (*p* = 0.063, *p* = 0.166) and variance homogeneity (*p* = 0.164, *p* = 0.457). One-way ANOVA showed that there are significant differences in both datasets on the 6th (F (2, 48) = 14, *p* < 0.0001) and 9th day (F (2, 47) = 80, *p* < 0.0001). At both time points, the mycelial extension length of three-conidia CFBs was significantly greater than the two-conidia CFBs (*p* < 0.0001) and significantly greater than the single conidium (*p* < 0.0001) (Figure 6).

## 4. Discussion

Conidia are the primary reproductive structures of asexual fungi and have evolved various adaptive strategies to survive in diverse and complex habitats [38,39]. Ensuring the survival of conidia in nutrient-poor and competitive habitats is a crucial evolutionary trend for fungi. Our study has revealed that the conidia fusion rate (CFR) decreases as the nutrient concentrations in the habitat increases (Figure 3 and Figure 4). This result is consistent with previous research and supports the idea that the formation of CF is closely related to the nutrient content of the habitat [21,23].

As the number of conidia in the conidia fusion body (CFB) increases, their germinated mycelium extends faster and more densely in nutrient-poor habitats such as 0% CMA and soil medium (Figure 5 and Figure 6). Similar to plant seeds, the nutrition for the growth of conidium into a mature germinator (the germinator with the function of absorbing nutrients from the habitats) mainly comes from the nutrients carried by the conidium. Therefore, the amount of nutrients carried by the conidia directly determines the development of the germinator (the elongation rate and density of mycelium). Once the conidium germinates into a mature germinator, the nutrients for their development mainly depend on mycelium absorbing from the habitat. Therefore, the elongation rate and density of mycelium determine the nutrient uptake efficiency of the germinator, which further determines whether the germinator can form a sustainable individual quickly. Obviously, larger conidia with more nutrients have more advantages in developing into a sustainable individual. CF enables the originally independent single and small conidium with fewer nutrients to form a CFB with more nutrients, allowing the mycelia germinated by the CFB to extend faster, farther, and more densely. This is further conducive to the CFB quickly searching for new nutrient sources in habitats with uneven nutrient distribution. Additionally, the dense mycelium increases the contact area with the environment, improves the nutrient absorption efficiency in nutrient-poor habitats, and enhances the resistance of conidia to nutrient-poor habitats. This in turn guarantees the survival rate of conidia and provides an advantage for the rapid development of the germinator. In this study, four out of the six species with CF were common species in many habitats (*Arthrobotrys oligospora*, *A. conoides*, *A. superba*, *A. musiformis*) [40,41,42,43]. Their widespread and common appearance may also be due to the formation of CF.

All 119 species of Orbiliomycetes nematode-trapping fungi (NTF) can be divided into three groups based on their conidia shape [34]. Arthrobotrys-shaped produce small conidia (usually < 30 × 15 μm) and are usually produced in large numbers; Monacrosporium-shaped produce larger conidia (usually > 30 × 15 μm) with a super-cell and usually less sporulation; Dactylella-shaped produce larger conidia (usually > 30 × 15 μm) with more septate (>3-septate) without the super-cell and less sporulation. Rubner proposed that the evolutionary trend of these three types of conidia is towards smaller size with fewer septa and more sporulation, whereby Dactylella-shaped conidia is the more primitive type which later evolved into Arthrobotrys-shaped and Monacrosporium-shaped conidia. All six species that produce CF screened out in this study belong to Arthrobotrys-shaped [34]. Aggregation growth and small conidia are the premises of mass production and efficient dissemination. However, the miniaturization of conidia is bound to reduce their nutrient reserves and survival ability in nutrient-poor habitats. While CF connects independent conidia (forming the CFB), multiply the nutrient reserves of CFB, thus guaranteeing their survival rate and enhancing their development advantages. Based on the above, we conclude that CF is an essential means to balancing the insufficient nutrient reserve caused by the miniaturization of conidia and is an important strategy to ensure the maintenance of the population size of related species.

The evolution of NTF is a critical issue in the study of carnivorous fungi [44]. Currently, mainstream evolutionary theory based on modern molecular biology focuses on the trapping structure as the main characteristic of the evolution of this group of fungi [27,28,29]. Yet it ignores the significance of conidia in evolution. However, the purpose of biological evolution can be simply reduced to better adaptation to the environment to ensure the survival and reproduction of the species. As the most important reproductive structure of asexual fungi, conidia should also have vital evolutionary significance in theory. Our study also suggests that the types of conidia and their accessory structures (CF) are important for the environmental adaptation and population maintenance of NTF. Therefore, conidia are also important in the evolution of this group of fungi and should be considered in future evolutionary studies.

The origin of multicellular organisms is one of the major unanswered questions in biological evolutionary research [45]. In 2021, Bernardes et al. demonstrated that under the stress of predators, *Chlamydomonas reinhardtii* (single-cell green algae) would connect and gather into cell clusters, thus reducing the probability of becoming consumed by predators, thereby increasing their survival rate. This structure can be steadily passed on to offspring under constant stimulation. This study suggests that single-cell connections congregating in groups to escape predation by predators may have contributed to the origin of multicellular organisms [46,47]. Similarly, our study found that the CF rate was inversely proportional to the nutrient content of the habitat. The formation of CF enhanced the adaptability of conidia to tolerate nutrient-poor habitats, and all strains of the six species that formed CF could form this structure (CF can be stably inherited). Therefore, the fusion of single conidium to adapt to the nutrient deficiency of their habitats may also be one of the reasons for the origin of multicellular organisms.

## Data Availability

The data that support the findings of this study are contained within the article.

## Supplementary Materials

The following supporting information can be downloaded at: https://www.mdpi.com/article/10.3390/jof9070755/s1, Figure S1: The source distribution map of the strains involved in this study; Table S1: Testing Nematode-trapping fungi.
