# Supplementary material for: Conidia Fusion: A Mechanism for Fungal Adaptation to Nutrient-Poor Habitats
Source: J Fungi (Basel). 2023 Jul 17;9(7):755. doi: 10.3390/jof9070755 (PMC10381365; doi:10.3390/jof9070755)
Supplement: Supplementary file 1 [file jof-09-00755-s001.zip › jof-2488817-supplementary.pdf]

Table S1: Testing Nematode-trapping fungi

Species name	Genera	Trap structure	Conidia type	Strain number
A. arthrobotryoides			Arthrobotrys-shaped	47
A. botryospora			Arthrobotrys-shaped	15
A. cladodes			Arthrobotrys-shaped	36
A. conoides			Arthrobotrys-shaped	237
A. eryuanensis			Arthrobotrys-shaped	3
A. javanica			Arthrobotrys-shaped	91
A. jindingensis			Arthrobotrys-shaped	2
A. jinpingensis			Arthrobotrys-shaped	2
A. Latispora			Arthrobotrys-shaped	6
A. musiformis			Arthrobotrys-shaped	350
A. oligospora			Arthrobotrys-shaped	457
A. polycephala			Arthrobotrys-shaped	3
A. pyriformis			Arthrobotrys-shaped	2
A. robusta			Arthrobotrys-shaped	1
A. shuifuensis			Arthrobotrys-shaped	3
A. superba			Arthrobotrys-shaped	276
A. tongdianensis	*Arthrobotrys*	Adhesive networks	Arthrobotrys-shaped	2
A. vermicola			Arthrobotrys-shaped	82
A. cystosporia			Monacrosporium-shaped	8
A. elegans			Monacrosporium-shaped	4
A. eudermata			Monacrosporium-shaped	43
A. fusiformis			Monacrosporium-shaped	1
A. gampsospora			Monacrosporium-shaped	3
A. gongshanensis			Monacrosporium-shaped	6
A. janus			Monacrosporium-shaped	1
A. lanpingensis			Monacrosporium-shaped	3
A. longiphora			Monacrosporium-shaped	8
A. luquanensis			Monacrosporium-shaped	3
A. luzhangensis			Monacrosporium-shaped	4
A. megalospora			Monacrosporium-shaped	3
A. microscaphoides			Monacrosporium-shaped	7
A. obovata			Monacrosporium-shaped	27
A. oudemansii			Monacrosporium-shaped	8
A. reticulata			Monacrosporium-shaped	44

A. scaphoides			Monacrosporium-shaped	54
A. sinensis			Monacrosporium-shaped	15
A. sphaeroides			Monacrosporium-shaped	49
A. thaumasia			Monacrosporium-shaped	91
A. xiangyunensis			Monacrosporium-shaped	187
A. zhaoyangensis			Monacrosporium-shaped	2
A. ultiformis			Dactylella-shaped	2
Dr. brochopaga	*Drechslerella*	Constricting rings	Arthrobotrys-shaped	3
Dr. dactyloides			Arthrobotrys-shaped	30
Dr. aphrobrocha			Monacrosporium-shaped	24
Dr. bembicodes			Monacrosporium-shaped	7
Dr. coelobrocha			Monacrosporium-shaped	31
Dr. daliensis			Monacrosporium-shaped	2
Dr. doedycoides			Monacrosporium-shaped	4
Dr. effusa			Monacrosporium-shaped	2
Dr. heterospora			Monacrosporium-shaped	1
Dr. stenobrocha			Monacrosporium-shaped	1
Dr. xiaguanensis			Monacrosporium-shaped	2
Da. cangshanensis	*Dactylellina*	Adhesive knobs	Monacrosporium-shaped	4
Da. drechsleri			Monacrosporium-shaped	54
Da. dulongensis			Monacrosporium-shaped	2
Da. ellispspora			Monacrosporium-shaped	71
Da. multiseptata			Monacrosporium-shaped	1
Da. parvicolla			Monacrosporium-shaped	6
Da. phymatopaga			Monacrosporium-shaped	1
Da. yushanensis			Monacrosporium-shaped	5
Da. illaqueata		Adhesive knobs and non-constricting rings	Monacrosporium-shaped	1
Da. sichuanensis			Monacrosporium-shaped	7
Da. cionopaga		Adhesive branches	Monacrosporium-shaped	3
Da. robusta			Monacrosporium-shaped	2
Da. gephyropaga			Monacrosporium-shaped	5
65 species	3 genera	5 types of trapping structures	3 types of conidia	2457 strains

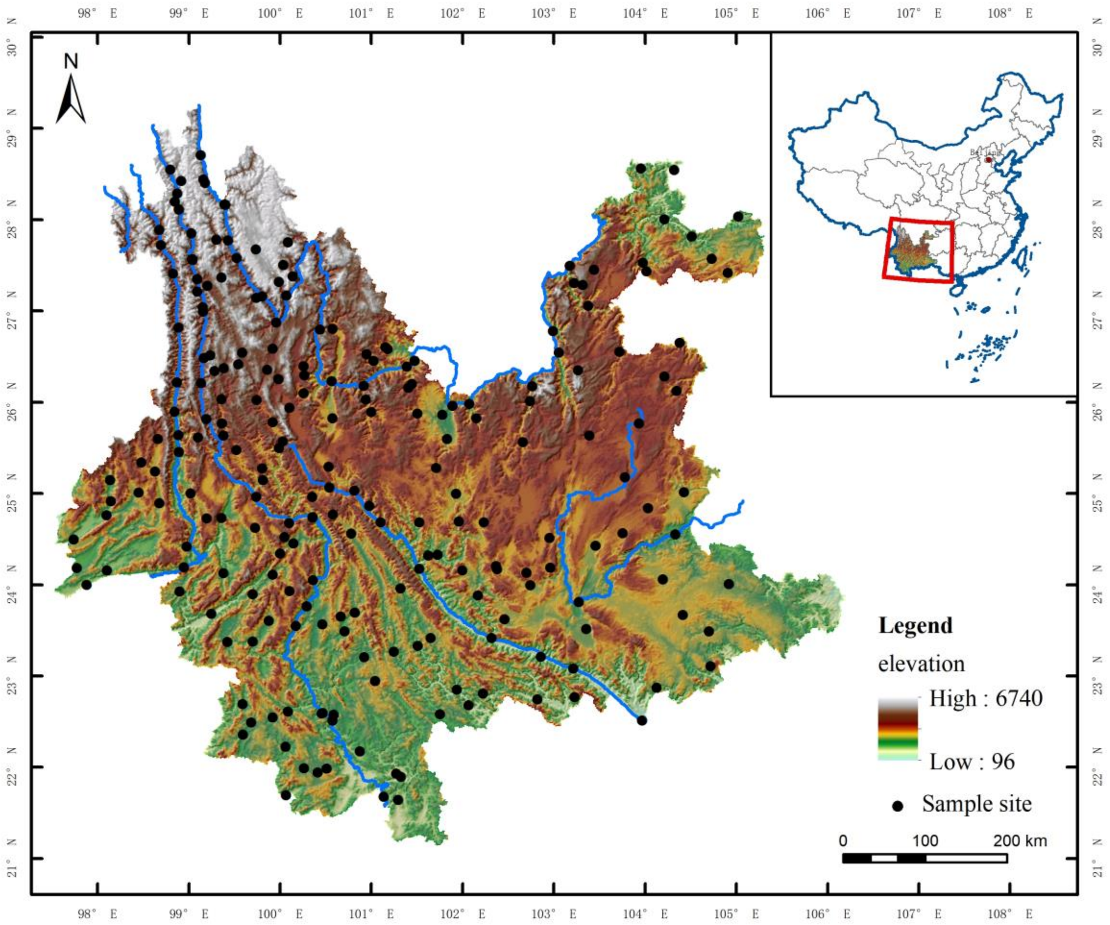

Figure S1. The source distribution map of the strains involved in this study.
